# Multifunctional Polymer Composite Materials

**DOI:** 10.3390/polym17121636

**Published:** 2025-06-12

**Authors:** Vineet Kumar, Md Najib Alam

**Affiliations:** School of Mechanical Engineering, Yeungnam University, 280, Daehak-ro, Gyeongsan 38541, Republic of Korea; vineetfri@gmail.com

## 1. Introduction

Recently, polymer composites have evolved from simple polymeric materials into advanced engineering systems capable of delivering multiple functionalities simultaneously [1,2]. This evolution has given rise to the concept of multifunctionality in polymer composite materials. They refer to the integration of diverse properties within a single system, making them suitable for a wide range of applications [3]. Multifunctionality is primarily enabled by the incorporation of functional fillers, such as carbon nanotubes [4], metal oxides [5], and various ceramic materials [6]. While many types of polymer matrices are available, elastomeric matrices are particularly favored. Among them, polyurethane [7], silicone rubber [8], and polydimethylsiloxane (PDMS) [9] are especially popular in energy-harvesting and -sensing applications. This choice is due to their inherent flexibility, stretchability, light weight, and ease of processing [10]. These characteristics facilitate the development of smart polymeric materials suitable for various applications. These applications are wearable sensors, soft robotics, energy-harvesting systems, vibration dampers, and advanced sensing platforms [11,12]. However, realizing true multifunctionality in polymer composites requires a careful balance of several critical properties. These include mechanical stiffness, uniform filler dispersion, and strong interfacial bonding between the filler and polymer matrix. Furthermore, maintaining mechanical integrity is essential, as it directly impacts the durability and intrinsic flexibility of the composite material [13].

However, achieving an optimal balance among these properties remains a significant challenge. To address this, filler functionalization is often employed to improve both filler dispersion and interfacial interactions between the filler and the rubber matrix [14]. Multifunctional polymer composites thus represent a transformative class of materials, like soft, adaptive, and intelligent, that are key enablers for next-generation technologies [15]. These advanced composites are specifically engineered for emerging applications such as human–machine interfaces, healthcare monitoring devices, and energy-harvesting systems. In light of these developments, this Special Issue aims to explore the multifunctionality of polymer composites in depth. It will highlight strategies for tailoring material properties to meet specific performance requirements. Therefore, they provide valuable insights and pathways for the practical implementation of these composites. Finally, the ability to control and optimize a wide range of mechanical, electrical, and structural properties enables the development of application-specific solutions. The following sections will review relevant literature and discuss how multifunctional polymer composites are being utilized across various domains. Finally, portable electronics, energy-harvesting technologies, and advanced sensing platforms are some important and targeted applications for this Special Issue.

## 2. Overview of Published Articles

Kumar et al. [16] report using silicone rubber-based composites and their multifunctionality as energy-harvesting systems. The authors use titanium carbide (TiC) and molybdenum disulfide (MoS_2_) to reinforce the matrix and improve the electrical conductivity of the composites. The results show that the output voltages were 3.5 mV (6 phr of TiC) and 6.7 mV (6 phr of MoS_2_). Similarly, Iyer et al. [17] designed and fabricated composites based on poly (ethylene oxide), and their multifunctional prospects for sodium ion batteries were demonstrated. Here, the authors develop a novel approach to designing the batteries by making them capable of operating at room temperature with high performance. The properties were fantastic, such as tensile strength of 32.1 MPa and an ionic conductivity of 1.01 × 10^−4^ S cm^−1^. Moreover, the battery shows high electrochemical stability and an energy density of 14.2 Wh kg^−1^. Therefore, this multifunctionality of the composites demonstrates a promising potential for developing high-performance batteries. Assadakorn et al. [18] use the Dunlop process to fabricate composites based on high-surface-area silica and natural rubber latex. The results show that the four types of silica particles were used, some with hydrophobicity and some with hydrophilicity. The hardness was tested, and it was 45 for non-silica foam rubber and 48 for hydrophilic fumed silica-based samples. Overall, the study concludes that the hydrophobic fumed silica was better than the hydrophilic ones in both fumed and precipitated silica particles in rubber composites. Another interesting review study by Malashin et al. [19] shows multifunctionalities in polymer composites. This review provides a special focus on new generation processing techniques like 3D and 4D printing. The review study further highlights that these emerging printing technologies are very promising in obtaining consistent composite samples. Finally, the paper summarizes the key takeaways, challenges, and future prospects of these techniques.

Sriani et al. [20] demonstrate ultrafiltration in whey protein separations. Here, the authors modify the polysulfone-based flat membrane with hydroxyapatite (HA) during fabrication using a wet-phase method. The results are interesting, as the permeability flux increases to 38% after adding 0.3 wt% of HA. Therefore, a simple and economical modification of the polysulfone membrane with HA resulted in improving permeability without sacrificing the separation efficiency. Therefore, this work provides an efficient route to obtain scalable membrane production for the high whey protein separation industry. Another study by Bril’ et al. [21] discusses the use of polymer composites for multifunctional applications like flexible electronics and electromagnetic compatibility. These composites are fabricated by mixing silver nanowires with a PET polymer matrix. As multifunctional applications, authors study the shielding efficiency for a 500–600 nm thick porous sample. The results show that the samples were 40 dB, while as the thickness increased to 3.1–4.1 μm, the efficiency increased to 85–90 dB. Finally, the results show that the samples were able to show their application for portable electronics and electromagnetic compatibility. Inna et al. [22] present an interesting study on possible staining and surface roughness of restored dental applications. This in vitro study provides a breakthrough in teeth restoration by micro-abrasion and resin infiltration (RIT). The study shows that RIT proves to be promising for higher durability with lower roughness. Fatkullin et al. [23] studied the switching of the polymer composites based on laser-induced graphene for multifunctional applications like tunable electronics. Moreover, the fabricated composites show strong potential for their use in energy storage, sensing, and bioelectronics. The new strategy proposed by the authors concludes that the tailoring of the polymer composite’s electrochemical properties with dynamic switching is possible. This aspect opens new pathways for flexible electronics with tunable properties and performance.

Repin et al. [24] study the multifunctionality of cellulose-based composite materials for water extraction from atmospheric air. A strong and porous composite system was developed that acts like a carrier for the precipitation of hygroscopic agents. The hygroscopic agents developed in this work were CaCl_2_ and 1-butyl-3-methylimidazolium chloride. Here, the use of 1-butyl-3-methylimidazolium chloride was found most promising for water absorption efficiency and recyclable potential. Plamadiala et al. [25] present a review article summarizing enhanced performance of polylactic acid (PLA) filaments in FDM. The polylactic acid was proffered because of its biodegradability and scalability due to easy processing. Finally, this study highlights the multifunctional use of PLA, including the potential challenges and processing conditions. Janmanee et al. [26] developed a biosensor based on polymer composites for the detection of dopamine (DA). The study shows that the DA imbalance is also associated with brain malfunction and results in diseases like Parkinson’s. The developed biosensor shows high-sensitivity selectivity in detecting DA content from 5 to 180 µM. Thus, the study supports that biosensing technology can be used to detect DA early and can avoid neurological disorders. Finally, Kumar et al. [27] provide a comprehensive review for studying physical activity monitoring through polymer composite sensors. This review study helps in understanding the early trends on the use of composite-based sensors for detecting physical activity from year 2020–2025. The composites are based on carbon nanomaterials as a conducting material and silicone rubber as the polymer matrix. The physical activities monitored in this work involve sensing running, cycling, and swimming. The electrical properties reviewed in this work include resistance, response time, and gauge factors of the strain sensor. Finally, the key challenges and future prospects of this work are summarized and reported.

## 3. Summary and Future Outlook

Although significant advancements have been made in the development of polymer composite materials, several critical challenges remain. These include difficulties in scalability, ensuring performance reliability, and achieving mechanical integration for long-term durability [28]. Addressing these issues requires focused research, particularly in the areas of filler dispersion, filler–polymer interfacial interactions, and material durability [29]. For instance, achieving uniform filler dispersion is essential for ensuring balanced and consistent performance in polymer composites. Poor dispersion often leads to filler aggregation, which reduces the effective interfacial area and hampers stress transfer during mechanical deformation [30]. Additionally, engineering the filler–matrix interface can substantially enhance stress distribution, charge transport, and overall durability. In contrast, weak interfacial bonding can cause performance degradation under mechanical, electrical, or thermal stress. Embedding multifunctional filler particles into high-performance functional devices also requires careful consideration of interface design, packaging, and interconnect architecture [31]. Fortunately, many of these challenges can be addressed through strategic approaches. For example, functionalizing filler particles can greatly improve dispersion and interfacial bonding, thereby unlocking the full performance potential of multifunctional composites [32]. Another promising route involves using hybrid filler systems, which can exhibit synergistic effects and deliver robust performance even at low filler loadings. Furthermore, there is a growing interest in bio-based polymers and biodegradable fillers, aimed at creating environmentally friendly multifunctional composites [33]. These sustainable materials are particularly well-suited for applications in transient electronics and green manufacturing. Looking ahead, multifunctional polymer composites are expected to play a pivotal role in numerous fields, including wearable electronics, soft robotics, biomedical devices, energy-harvesting systems, and structural health monitoring [34]. Overall, the future of composite-based sensors and devices appears highly promising, offering a sustainable, resilient, and versatile pathway toward green energy and next-generation sensing technologies.
